# Depressive symptoms as predictors of sexual experiences among very young adolescent girls in slum communities in Nairobi, Kenya

**DOI:** 10.1080/02673843.2020.1756861

**Published:** 2020-05-02

**Authors:** Beatrice W. Maina, Benedict O. Orindi, Jane Osindo, Abdhalah K. Ziraba

**Affiliations:** a African Population and Health Research Center, Nairobi, Kenya; b School of Public Health, University of the Witwatersrand, Johannesburg, South Africa; c Department of Public Health and Primary Care, Leuven Biostatistics and Statistical Bioinformatics Centre, KU Leuven, Leuven, Belgium

**Keywords:** Adolescents, sexual behaviours, depression, mental health, HIV, latent class analysis

## Abstract

Mental health issues are a predisposing factor for HIV acquisition. We examined the association between depressive symptoms and patterns of sexual experience among girls aged 10–14 years living in Korogocho and Viwandani slums in Nairobi, Kenya. We analysed data collected in 2017 from a random sample of 606 girls. Using Latent Class Analysis, we modelled patterns of sexual experiences and used multivariable regression analysis to determine the association between self-reported depressive symptoms and sexual experiences. Seven in ten girls reported at least one symptom of self-reported depression in the past 12 months. About 13% of girls had had a sexual experience, resulting in two patterns of sexual experience – naïve and experienced. Girls reporting depressive symptoms were more likely to be sexually experienced. Sexual and reproductive health programs targeting adolescent girls should consider including intervention packages that address mental health conditions such as depression.

## Introduction

The World Health Organization defines mental health as ‘*a state of well-being in which every individual realizes his or her own potential, can cope with the normal stresses of life, can work productively and fruitfully, and is able to make a contribution to her or his community*’ (World Health Organization 2014). Most mental health problems in developing countries go untreated (Audet et al., 2017) and contribute significantly to the burden of disease among young people (World Health Organization, 2014a). This study looks at depression, as a mental health manifestation, and examines the association between self-reported depressive symptoms and sexual experiences among very young adolescent girls (VYAG), also referred to as early adolescent girls, in two urban slums in Nairobi, Kenya.

Although past studies have reported the association between depression and sexual experiences among young people in sub-Saharan settings, especially in the context of HIV (Audet et al., 2017; Cucchiaro & Dalgalarrondo, 2007; Kamndaya et al., 2017; Musisi et al., 2014), there is limited literature focusing on VYAG, yet, their developmental stage elevates their risk for depression and sexual risk behaviours (Bennett & Bauman, 2000; Kaltiala-Heino et al., 2003; Kastbom et al., 2015) as they navigate pubertal changes (Dixon-Mueller, 2009; L’Engle & Jackson, 2008). Sexual expressions and behaviours begin to manifest in early adolescence (the period between 10 and 14 years) as pubertal maturation sets in (Kågesten et al., 2018). While romantic and, to some extent, sexual experiences in early adolescence are considered normative in some settings, especially in developed regions (Tolman & McClelland, 2011), in many African contexts, sexual experiences and relationships in early adolescence are disapproved (Akers et al., 2011). Yet, recent studies indicate high rates of sexual experiences among very young adolescents in sub-Saharan Africa (Beguy et al., 2013; Kågesten et al., 2018), pointing to a need for more research on drivers of early sexual activities in this region.

Inarguably, depression may inhibit VYAG’s ability to assess risks associated with early sexual activity or undertake mitigation strategies such as condom use (Kamndaya et al., 2017; Lundberg et al., 2011; Musisi et al., 2014). Majority of available literature on depression and sexual experiences among adolescents either focus on older adolescents ages 15–19 or does not disaggregate findings by age, despite the differences in adolescent developmental stages (Espinosa-Hernandez & Vasilenko, 2015; Mazzaferro et al., 2006; Musisi et al., 2014; Othieno et al., 2015). In a study of middle-school Mexican adolescents aged 12–19 years, Espinosa-Hernandez and Vasilenko (2015) found that adolescents who were in committed sexual or romantic relationships had the highest level of depressive symptoms. Another study investigating the association between psychosocial factors with risky sexual behaviours among adolescents and young women aged 14–25 years found that depression and stress were associated with high-risk sexual behaviours (Mazzaferro et al., 2006) including unprotected sex, sexual violence and multiple sexual partnerships with implications on an individual’s vulnerability to HIV infection (Lane et al., 2008; Moore et al., 2007). It is also probable that young people experiencing depressive symptoms may engage in risky sexual behaviours as a way of exerting control over their life or as a diversion, a way to release stress or tension or as an affection seeking strategy. On the flip side, sexual experiences can expose one to depression especially in young adolescents who might not be prepared to confront unexpected social pressures such as rejection, dropping out of school, financial needs, sexually transmitted illnesses and unintended pregnancies among other challenges (Foshee et al., 2013; Turner et al., 2006).

Contextual factors, such as high levels poverty, violence, including domestic, sexual and physical violence, alcohol and substances use, and adverse childhood experiences, that are common in urban slums (African Population and Health Research Center, 2014; Cucchiaro & Dalgalarrondo, 2007; Kamndaya et al., 2017; Schilling et al., 2007), increase the likelihood of young people engaging in risky sexual behaviours (Abdulraheem & Fawole, 2009; Kabiru et al., 2010; Madise et al., 2012) as well as elevated risk for depression (Kamndaya et al., 2017; Magidson et al., 2017; Schilling et al., 2007). However, there has been little focus on exploring the linkages between depression and sexual behaviours among VYAG, whom, early sexual activity could have detrimental effects on their health and wellbeing across the life course. Additionally, existing literature on sexual activity in adolescence focuses largely on sexual intercourse, yet, young people have been found to engage in other non-penetrative sexual activities that are important in understanding sexual risk (Kågesten et al., 2018; Tolman & McClelland, 2011)

Against this backdrop, we describe sexual behaviours among VYAG aged 10–14 years in two urban slums in Nairobi, Kenya and examine the association between these sexual behaviours and self-reported depressive symptoms.

## Methods

### Study design, setting and sample

We analysed round one interview data collected in 2017 from a random sample of VYAG selected for the impact evaluation of DREAMS (Determined, Resilient, Empowered, AIDS-free, Mentored, and Safe women) initiative in Korogocho and Viwandani slums in Nairobi, Kenya. The DREAMS interventions are a package of individual, household-level and community-wide interventions aimed at reducing the vulnerability of adolescent girls and young women to acquiring HIV (Saul et al., 2018). The full impact evaluation study protocol has been published elsewhere (Birdthistle et al., 2018). In brief, participants were recruited from the Nairobi Urban Health and Demographic Surveillance System (NUHDSS), a longitudinal platform that has been running in Korogocho and Viwandani slums since 2002 (African Population and Health Research Center, 2019; Beguy et al., 2015). The survey tool was adapted from a toolkit developed, validated and piloted in the context of the Global Early Adolescent Study, an international study focusing on factors that predispose very young adolescents to poor health outcomes including sexual risk (Global Early Adolescent Study, 2014). Data were available on 606 girls (Korogocho, n = 323; Viwandani, n = 283) aged 10–14 years.

### Procedures

Data were collected electronically using face-to-face interviews by 14 experienced and well-trained female field interviewers who were also well conversant with the study area. The interviews were conducted in secure, private locations at a local school or NUHDSS field office during weekends and holiday breaks (when school was not in session). Transport reimbursement was provided for VYAG who required transport to access the interview site. All participants were provided with a snack (mainly a loaf of bread and a packet of milk) during the interview. The tool which was also translated into Swahili was pre-tested and necessary adjustments made prior to the main survey. Interviews were conducted in either Swahili or English depending on the preference of the participant. The study was approved for ethical and scientific merit. All parents and adolescent girls provided informed consent and assent, respectively, prior to participating in the study.

### Measures

#### Outcome variable

The outcome of interest was sexual experience (or behaviour), assessed by latent class membership using 11 sexual experience items. The items are listed in Box 1. The item ‘*Today which statement best describes you?’*, which measured relationships status had 8 response categories of ‘Never been in romantic relationship’ (=0), ‘Not currently in romantic relationship, but have had a boyfriend before’ (=1), ‘Have more than one girlfriend’ (=2), ‘Have more than one boyfriend’ (=3), ‘Have a girlfriend’ (=4), ‘Have a boyfriend’ (=5), ‘I’m engaged to be married’ (=6), ‘I’m married’ (=7). We transformed this item into ‘yes’ (=1) if the girl had ever been in a romantic relationship (i.e., options 1–7), and ‘no’ (=0) otherwise. The remaining 10 sexual experience items were dichotomized into ‘no’ (=0) and ‘yes’ (=1)

**Box 1.** The 11 items used to measure sexual experiences among VYAG.

Items
1. Today which statement best describes you?
2. Have you ever spent time alone with someone you were in love with alone?
3. Ever held hands with someone you were in love with?
4. Ever hugged or cuddled with someone you were in love with?
5. Ever kissed or been kissed by someone on the lips or with tongue
6. Ever flirted with someone using a phone, email, or social media? ꝉ
7. Ever sent a sexual picture of yourself to someone using the phone, email, social media? ꝉ
8. Ever touched another boy or girls’ private parts or been touched?
9. Ever had sexual intercourse?
10. Ever had oral sex?*
11. Ever had anal sex?*

* Excluded from the analysis due to low frequencies. Also to note, VYAG who had experienced oral or anal sex had also experienced sexual intercourse. ꝉ Combined into a single item due to very low frequencies and similarity in meaning; namely, ‘Ever flirted with or sent sexual picture of yourself to someone using a phone, email, or social media?’

#### Moderating variable

The moderating variable was actual participation in DREAMS activities – defined by participation in at least one of the four primary DREAMS activities specific to the 10-14-year-olds. That is, HIV testing and counselling services (HTS) or linkage to HTS, school-based HIV & violence prevention, financial capability or literacy training, and social asset building (SAB). Important to note here is that the data used in this analysis were collected in the early stages of DREAMS implementation and hence the likelihood of any significant effects attributable to the intervention is negligible.

#### Predictor variable

The main predictor variable was self-reported depression measured using a depression scale with 6 items (Table 3). Each item had 5 response categories of ‘Agree a lot’ (=1), ‘Agree a little’ (=2), ‘Neither agree, nor disagree’ (=3), ‘Disagree a little’ (=4), ‘Disagree a lot’ (=5). Other than the item of ‘*In general, I see myself as a happy person*’, the items were reversed so that, for all the items, a higher score indicates high experience of the depressive symptom. This depression scale was piloted in 2015 among 214 VYAG aged 11–14 years in Korogocho slums in the context of the Global Early Adolescent Study. The scale items had a Cronbach’s coefficient α of 0.8533, showing a satisfactory internal consistency.

**Table 1.** Distribution of study participants by selected characteristics*.

	All respondents	non-DREAMS participants		DREAMS participants	
Variable	Number of girls (N)	Number of girls (n)	Percent of girls (row%)	Number of girls (n)	Percent of girls (row%)
DREAMS participation					
No	343	343	100	-	-
Yes	263	-		263	100
Age (years)	Mean = 12.07, SD = 1.30	Mean = 11.90, SD = 1.34		Mean = 12.28, SD = 1.22	
Site					
Korogocho	323	135	41.8	188	58.2
Viwandani	283	208	73.5	75	26.5
Religion					
Christian	534	307	57.5	227	42.5
Muslim	61	28	45.9	33	54.1
Other	11	8	72.7	3	27.3
Ethnicity					
Kikuyu	195	109	55.9	86	44.1
Luo	115	53	46.1	62	53.9
Luhya	92	54	58.7	38	41.3
Kamba	96	59	61.5	37	38.5
Kisii	34	31	91.2	3	8.8
Somali	55	25	45.5	30	54.5
Other	19	12	63.2	7	36.8
Migration status					
Not born within DSA	229	155	67.7	74	32.3
Born in DSA	377	188	49.9	189	50.1
Pubertal maturation					
Pre-pubertal	144	92	63.9	52	36.1
Pubertal	462	251	54.3	211	45.7
Body comfort	Mean = 18.71, SD = 3.15	Mean = 18.72, SD = 3.32		Mean = 18.71, SD = 2.92	
Adverse childhood experiences (ACES)					
No experience	89	49	55.1	40	44.9
1-2 ACES	264	133	50.4	131	49.6
3-4 ACES	140	88	62.9	52	37.1
5+ ACSES	113	73	64.6	40	35.4
Ever experienced bullying, GBV and IPV					
No	377	209	55.4	168	44.6
Yes	229	134	58.5	95	41.5
Ever perpetrated bullying, GBV and IPV					
No	462	246	53.2	216	46.8
Yes	144	97	67.4	47	32.6
Substance					
No	597	339	56.8	258	43.2
Yes	9	4	44.4	5	55.6

*SD is standard deviation; DSA is demographic surveillance area; GBV is gender-based violence; IPV is intimate partner violence.

**Table 2.** Frequencies and average score for indicators used to measure self-reported depression.

	Response categories frequencies					
Indicators for depresion	1	2	3	4	5	Median (IQR)
In general, I see myself as a happy person	527	54	6	8	11	1 (1–1)
I blame myself when things go wrong*	290	67	10	93	146	2 (1–4)
I worry for no good reason*	395	52	5	76	78	1 (1–4)
I am so unhappy I can’t sleep at night*	458	58	5	42	43	1 (1–1)
I feel sad*	433	49	6	71	47	1 (1–2)
I am so unhappy I think of harming myself*	521	38	6	18	23	1 (1–1)
Average score						1.17
Depression sum-score						Mean = 10.47, SD = 4.53

*Response categories were reversed such that 1 = ‘Disagree a lot’, 2 = ‘Disagree a little’, 3 = ‘Neither agree, nor disagree’, 4 = ‘Agree a little’, 5 = ‘Agree a lot’

**Table 3.** Sexual experiences among very young adolescent girls by participation in DREAMS activities.

	Percentage of girls reporting item		
Item	Combined (n = 606)	Never participated in DREAMS (n = 343)	Participated in DREAMS (n = 263)
1. Have been in a romantic relationship	10.6	11.1	9.9
2. Have you ever spent time alone with someone you were in love with alone?	8.4	9.3	7.2
3. Ever held hands with someone you were in love with?	8.8	9.6	7.6
4. Ever hugged or cuddled with someone you were in love with?	5.0	5.8	3.8
5. Ever kissed/been kissed by someone on the lips or with tongue	1.8	2.0	1.5
6. Ever flirted with or sent sexual picture of yourself to someone using a phone, email, or social media?	2.2	2.0	2.3
7. Ever touched another boy or girls’ private parts or been touched?	1.2	1.2	1.1
8. Ever had sexual intercourse?	2.0	2.0	1.9
Prevalence of sexual experience	12.9	13.4	12.2

#### Control variables

Four control variables were considered. They included 1) adverse childhood experiences measured using 13 items on a 3-point Likert scale of 1 ‘never’, ‘sometimes’ and ‘often’; 2) violence victimization, 3) violence perpetration and 4) alcohol and substance abuse. Forms of violence included in victimization and perpetration were bullying and teasing, physical violence, and intimate partner violence. Additionally, we controlled for socio-demographic characteristics including age, religion, ethnicity, parental survivorship, migration status, pubertal maturation and body comfort.

### Analysis

First, we summarized the distribution of the participants across all variables. We also estimated proportions of girls reporting a sexual experience. These proportions were compared across those who did and did not participate in DREAMS.

Next, on the basis of the sexual experience items, we identified the categories of sexual behaviours using latent class analysis (LCA). LCA is a statistical technique used to group individuals into classes (categories) of unobserved (latent) variables on the basis of the responses from multivariate data (Collins & Lanza, 2010). Specifically, LCA are part of a larger group of models known as finite mixture models (McLachlan et al., 2019; Muthén, 2002). Formally, let j=1,…,J be the observed (manifest) variables with $r_{j}=1,\ldots ,R_{j}$ the corresponding response categories. Y is the vector response of patterns such that y represents a particular response pattern; and L represents the categorical latent variable with $\left(c=1,\ldots ,C\right)$ latent classes. Then, the probability of observing a particular vector of responses is given by the expression,

(1) $$P\left(Y=y\right)=\overset{C}{\underset{c=1}{\sum }}\gamma_{c}\overset{J}{\underset{j=1}{\prod }}\overset{R_{j}}{\underset{r_{j}=1}{\prod }}\rho_{j,r_{j}|c}^{I\left(y_{j}=r_{j}\right)}$$

where $\gamma_{c}$ is the probability of membership in latent class c (i.e., $\gamma_{c}=P\left(L=c\right),$ $\rho_{i|c}$ the probability of response i to item j conditional on membership in latent class c, and $I\left(y_{j}=r_{j}\right)$ is an indicator function that equals 1 when the response to item $j=r_{j}$ and 0 otherwise. We fitted a series of models with an increasing number of latent classes, and the appropriate number of classes was chosen based on Akaike’s Information Criteria (AIC), sample-size-adjusted Bayesian Information Criteria (ssBIC), log likelihood, and the bootstrapped parametric likelihood ratio test (LRT). The bootstrapped parametric LRT has been suggested in the literature to be more reliable than the Lo-Mendell-Rubin adjusted LRT. It compares the model with C latent classes to a model with $\left(C-1\right)$ classes. A significant p-value is in support of a model with C classes over a model with $\left(C-1\right)$ classes. In addition, we also considered our theoretical expectations and the interpretability of those classes (Collins & Lanza, 2010; Muthén & Muthén, 1998–2015). We then assessed differential effect of DREAMS (i.e., whether the latent subgroups appear similarly among DREAMS and non-DREAMS participants) by 1) first establishing whether the number of latent classes is identical across the groups by fitting single group models in each group separately; and 2) multigroup LCA, with DREAMS participation as the grouping variable. Any dependence on the grouping variable in the measurement model indicates lack of measurement equivalence, i.e. that the measurement properties of at least one item vary across the groups.

Finally, using latent class regression (LCR) obtained by extending expression (1) to include covariates (Collins & Lanza, 2010), we adopted a two-step approach to examine the characteristics of the sexual experience typologies identified above. First, simple models were fitted for each covariate separately and those found to be significant at P < 0.10 were included in a multivariable model, and tested at P < 0.05 in the final model. With the first latent class as the reference, adjusted odds of being in a specific class were estimated. Data management and descriptive analyses were performed using STATA v15 (StataCorp, College Station, TX). Latent class analyses was performed using Mplus v7.4 (Muthén & Muthén, 1998–2015).

## Results

### Sample description

Of 606 girls, 43% (*n* = 263) had participated in at least one of the four DREAMS primary activities meant for the 10 – 14-year-olds. Their average age was 12.07 (SD = 1.30). The median response category for all depression indicators was 1 (‘Agree a lot’), except for the item ‘*I blame myself when things go wrong’*, which also exhibited the highest variability (Table 3). Table 1 summarizes characteristics of the VYAG girls, combined and by their participation in DREAMS activities. In general, there were some variations across the characteristics. About half of the girls were from Korogocho and six in ten girls were born within the two study sites. A higher proportion of participants was from Kikuyu ethnic group (32%) and about 76% had started experiencing pubertal changes.

### Self-reported depressive symptoms among very young adolescent girls

Using top-two box scoring (i.e., ‘Agree a little’ and ‘Agree a lot’), about 60% (n = 361) of girls reported at least one depressive symptom. Table 2 presents the frequencies and average score for indicators used to measure self-reported depression status. The mean (SD) depression sum-score was 10.47 (4.53).

### Prevalence of sexual experiences among very young adolescent girls

Table 3 shows percentage of girls reporting to have ever had a sexual experience, combined and by participation in DREAMS. Overall, about 13% had a sexual experience. This proportion was 13% and 12% among non-DREAMS and DREAMS participants, respectively. A higher proportion (11%) of girls reported having been in a romantic relationship. Romantic and sexual activities that girls reported to have engaged in with someone they were in love with included holding hands (9%), spending time alone (8%), and hugging or cuddling (6%), kissing or being kissed (2%), touching another boy or girls’ private parts or been touched (1%), and sexual intercourse (2%). It can be seen from Table 3 that these proportions were generally greater among non-DREAMS participants than DREAMS participants.

### Patterns of sexual experiences among very young adolescent girls

Supplementary Table S1 shows goodness-of-fit indices for latent class models with one, two, three, and four latent classes fitted to the entire VYAG sample. The bootstrapped parametric LRT indicated that a 2-class model was preferable to a single-class model (P < 0.001), and that three classes were, in turn, better than two classes (P < 0.001). A four-class solution was not interpretable. The other fit indices also indicated that the three-latent class solution represented the data best. However, we experienced computational challenges with a 3-class model due to low frequency in the third class (n = 4, 0.7%). Thus, we considered a 2-class model. The two classes were clearly interpretable and were in line with our theoretical expectations. Of the 606 girls, about 91% (n = 549) were in the first class and 9% (n = 57) in the second class.

**Figure 1.**
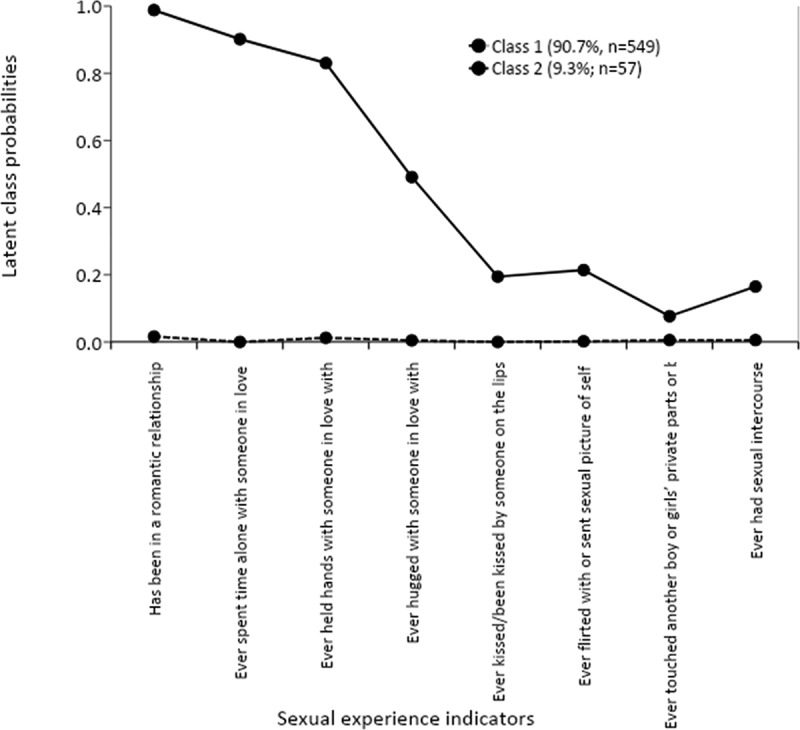
Profile plots indicating probabilities of the two-latent categories of sexual experiences among VYAG girls in Nairobi slums.

Figure 1 presents the profile plots showing the probability of a ‘yes’ response to an item, given that the girl belongs to a particular sexual experience class. Class 1 consisted of girls with almost zero probabilities of reporting any sexual experience activity. As such, we labelled class one as ‘Naive’. Class 2 consisted of girls with higher chances of reporting they had been in a romantic relationship (97%), had ever spent time alone with someone they were in love with alone (90.2%), ever held hands with someone they were in love with (82%), ever hugged or cuddled with someone they were in love with (49%). Girls in this class were also likely to report to have kissed or been kissed by someone on the lips or with tongue, flirted with or sent sexual picture of themselves to someone using a phone, email, or social media, touched another boy or girls’ private parts or been touched, or had ever had sexual intercourse. We labelled this class as ‘experienced’.

The Supplementary Table S1 also shows the goodness-of-fit indices for non-DREAMS and DREAMS participants, respectively. The indices support two- and three-latent-class models, but for reasons explained above for the entire VYAG sample, a 2-class model was preferred. Multigroup LCA showed no indications of lack of measurement equivalence between DREAMS and non-DREAMS participants.

### Sexual experiences and self-reported depression among very young adolescent girls

**Figure 2.**
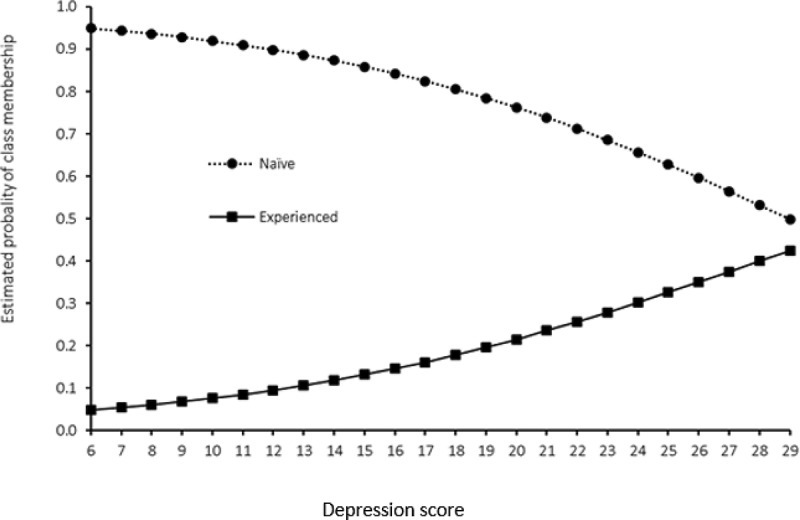
Probability of class membership by depression score estimated using LCR model.

Table 4 summarizes the odds of being in the experienced category relative to the naïve category with respect to covariates from the LCR model. Self-reported depression was significantly associated with sexual experience among VYAG living in Korogocho and Viwandani slums. After adjusting for body comfort, experience of violence, and substance abuse, the odds of being in the experienced category than in the naïve category increased with an increase in the depression score (light: aOR = 1.11, 95%CI 1.05–1.17). Figure 2 presents this pictorially. It shows that while girls with lower self-reported depression scores had greater probability of being in the naïve category, the probability of being in that category decreased for girls with high self-reported depression scores. Girls who had ever experienced violence were twice as likely to be in the sexually experienced category (aOR = 2.09, 95%CI 1.16–3.75) than naïve category.

**Table 4.** Estimated class membership odds ratios (95% CI) using multivariable LCR model*.

	Experienced vs. Naïve	
Covariates	aOR (95%CI)	P-value
Depression	1.11 (1.05–1.17)	<0.001
Body comfort	0.95 (0.87–1.04)	0.267
Ever experienced bullying, GBV and IPV		
No	Ref.	
Yes	2.09 (1.16–3.75)	0.014
Ever used alcohol/smoked/other drugs		
No	Ref.	
Yes	2.33 (0.44–12.29)	0.320

*aOR is adjusted odds ratios; 95%CI is 95% confidence intervals; GBV is gender-based violence; IPV is intimate partner violence.

## Discussion

This paper explores the relationship between self-reported depression and sexual experiences among adolescent girls aged 10–14 years living in two urban informal settlements in Nairobi, Kenya. First, we examined the prevalence of sexual experiences among this population and found that about 13% of girls had ever experienced a penetrative or non-penetrative sexual activity, a finding similar to other few studies that have focused on sexual experience in early adolescence in similar settings. For instance, Kabiru et al. (2010) and Kågesten et al. (2018) found that though majority of very young adolescents had not engaged in sexual intercourse, they were engaged in other non-penetrative sexual activities such as kissing, holding hands, touching or fondling and spending time together. While this indicates that most of the girls aged 10–14 years are yet to engage in sexual intercourse, there is a considerable proportion of girls who have had other sexual experiences. The large proportion of VYAG who have not had sexual experiences justifies the need and an opportunity to intervene with positive behaviour models before norms are translated into behaviours.

Secondly, we examined the prevalence of self-reported depression among the study participants. About 60% of girls had experienced at least one self-reported depressive symptom, an indication that depression, and by extension, mental health issues, are prevalent among VYAG in the study setting, and a public health concern that needs to be addressed. Though few past studies have explored mental health among this population, a study conducted in the slums of Kampala, Uganda found that nearly one in four youth aged 12–18 years reported suicidal ideation in the year preceding the study (Culbreth et al., 2018). Another study conducted in Western Cape, South Africa found that 17% of children and adolescents reported mental health disorders (Kleintjes et al., 2006). Urban informal settlements are characterized by extremely high social, environmental and physical risks (Beguy et al., 2013; Kabiru et al., 2010; Kågesten et al., 2018) which are likely to predispose young people to mental health risks.

Lastly, we examined the association between self-reported depressive symptoms and sexual experiences among VYAG. Our findings indicate an independent association between depressive symptoms and sexual experience which may increase vulnerability to HIV infection (R. Le et al., 2002). Depression, like other mental health conditions, may result in poor impulse control, impaired decision-making, and inability to assess risk increasing the likelihood of engaging in early or risky sexual behaviours (Havens & Mellins, 2008), with implications on HIV infections. Other studies have found a positive association between depression and sexual experiences (Agardh et al., 2012; Kaltiala-Heino et al., 2003; Kosunen et al., 2003; Lundberg et al., 2011; Mazzaferro et al., 2006; Schilling et al., 2007; Wilson et al., 2010). For instance, a study among adolescents aged 14–16 years found that self-reported depression was associated with experience of sexual intercourse (Kaltiala-Heino et al., 2003).

Thus, our findings shows the importance of orienting focus on mental health while addressing sexual behaviours among VYAG. Though we did not capture HIV status among the participants, our findings are important for HIV programming especially in the era when adolescents are beginning to engage in sexual activity at an early age (Kabiru et al., 2010; Kågesten et al., 2018; Marston et al., 2013). While existing literature demonstrates a high prevalence of depressive symptoms among adolescents living with HIV/AIDS (Lam et al., 2007), it is important to recognize the low rates of HIV testing among adolescents. For instance, in 2018, only about 19% and 14% of girls and boys, respectively, in Eastern and Southern Africa tested for HIV and received results (UNICEF, 2019).

Our findings show that girls who had experienced violence were more likely to be in the sexually experienced compared to the naïve category. While evidence exists on violence victimization in romantic and sexual relationships among young people (Brown et al., 2009; Otwombe et al., 2015) studies have also drawn linkages between violence victimization and depression (Kamndaya et al., 2017; Patel et al., 2007; M. T. H. Le et al., 2016), and in turn, our findings show that depressive symptoms are associated with sexual experiences among the VYAG.

### Implications

Given the contexts that young people in urban slums live in, mental health issues and associated factors must be tackled with urgency before they reverse the gains achieved in reducing HIV incidence in sub-Saharan Africa.
