# Supplementary material for: Depressive symptoms as predictors of sexual experiences among very young adolescent girls in slum communities in Nairobi, Kenya
Source: Int J Adolesc Youth. 2020 May 2;25(1):836–48. doi: 10.1080/02673843.2020.1756861 (PMC7254498; doi:10.1080/02673843.2020.1756861)
Supplement: Supplemental Material [file RADY_A_1756861_SM4158.docx]

**Depressive symptoms as predictors of sexual experiences among very young adolescent girls in urban slum communities in Nairobi, Kenya**

Supplementary Table S1. Selecting number of latent classes for sexual experiences among very young adolescent girls

| Number of latent classes | AIC | ssBIC | Log-likelihood | Bootstrapped parametric LRT p-value |
| --- | --- | --- | --- | --- |
| Entire sample (n=606) |  |  |  |  |
| 1 | 1802.7 | 1812.6 | -893.4 | _ |
| 2 | 1064.1 | 1085.1 | -515.1 | <0.001 |
| 3 | 1035.0 | 1067.0 | -491.5 | <0.001 |
| 4 | Solution not interpretable | | |  |
| Non-DREAMS participants (n=343) only | | | |  |
| 1 | 1085.6 | 1091.0 | -534.8 | _ |
| 2 | 631.2 | 642.5 | -298.6 | <0.001 |
| 3 | 613.2 | 630.5 | -280.6 | <0.001 |
| 4 | 623.0 | 646.3 | -276.5 | 0.6667 |
| DREAMS participants (n=263) only | | | |  |
| 1 | 729.6 | 732.8 | -356.8 | _ |
| 2 | 459.0 | 465.9 | -212.5 | <0.001 |
| 3 | 458.0 | 468.5 | -203.0 | 0.030 |
| 4 | 463.2 | 477.2 | -196.6 | 0.1923 |
